# LncRNA OIP5‐AS1 Promotes Glomerular Mesangial Cell Senescence by Interacting With ELAVL1 to Upregulate HMGB1

**DOI:** 10.1002/cbf.70311

**Published:** 2026-09-20

**Authors:** Zhiwei Wu, Na Xue, Huijun Cheng, Hengcai Hu, Li Sun, Zhenxin Ren

**Affiliations:** ^1^ College of Biological Science and Technology Yili Normal University Yining Xinjiang Uygur Autonomous Region P. R. China; ^2^ Xinjiang Key Laboratory of Lavender Conservation and Utilization Yining Xinjiang Uygur Autonomous Region P. R. China; ^3^ School of Basic Medical Sciences Gansu University of Chinese Medicine Lanzhou Gansu Province P. R. China

**Keywords:** ELAVL1, glomerular mesangial cell, HMGB1, OIP5‐AS1, senescence

## Abstract

Glomerular mesangial cell (GMC) senescence contributes to the progression of various age‐related kidney diseases. However, the molecular mechanisms driving GMC aging remain poorly understood. OIP5‐AS1, a long non‐coding RNA (lncRNA), has been implicated in cellular senescence in other systems, but its role in renal aging remains unclear. This work aimed to elucidate the functional role and underlying mechanism of OIP5‐AS1 in GMC senescence. A D‐galactose (D‐gal)‐induced senescence model was established in both mouse kidneys and SV40‐MES13 mesangial cells. RT‐qPCR was performed to quantify the levels of OIP5‐AS1, ELAVL1, and HMGB1, while CCK‐8 assays assessed cellular metabolic activity. Cellular senescence was evaluated using SA‐β‐gal staining, and the expression of senescence‐associated proteins (p16, p21, p53) was quantified by Western blot analysis. Levels of SASP‐related cytokines, including IL‐6, IL‐1β, TNF‐α, and IL‐18, were measured via ELISA. To explore the underlying mechanism, RNA pull‐down and RIP assays were performed to investigate the interactions among OIP5‐AS1, ELAVL1, and HMGB1, while actinomycin D assays were used to evaluate HMGB1 mRNA stability. OIP5‐AS1 expression was markedly elevated in renal tissues and GMCs following D‐gal‐induced senescence. Knockdown of OIP5‐AS1 alleviated cellular senescence. Mechanistically, OIP5‐AS1 directly interacted with ELAVL1, thereby stabilizing HMGB1 mRNA and promoting HMGB1 expression. Rescue experiments further confirmed that ELAVL1 and HMGB1 mediated OIP5‐AS1‐induced GMC senescence. LncRNA OIP5‐AS1 promotes GMC senescence by interacting with ELAVL1 to upregulate HMGB1.

## Summary

- OIP5‐AS1 expression is upregulated in d‐gal‐induced renal and mesangial cell senescence, and inhibition of OIP5‐AS1 suppresses glomerular mesangial cell senescence.
- OIP5‐AS1 interacts with ELAVL1 to stabilize HMGB1 mRNA.
- The OIP5‐AS1/ELAVL1/HMGB1 axis promotes glomerular mesangial cell senescence.

**Abbreviations**
DAMP: damage‐associated molecular pattern
d‐gal: d‐galactose
ELAVL1: embryonic lethal abnormal vision‐like RNA‐binding protein 1
GDP: Guiqi Dingnian prescription
GMCs: glomerular mesangial cells
HMGB1: high mobility group box 1
lncRNA: long non‐coding RNA
OIP5‐AS1: Opa interacting protein 5 antisense RNA 1
RIP: RNA immunoprecipitation
RT‐qPCR: reverse transcription quantitative polymerase chain reaction
SASP: senescence‐associated secretory phenotype
SA‐β‐gal: senescence‐associated β‐galactosidase
sh‐RNA: short hairpin RNA
WB: western blot

## 1 Introduction

Glomerular mesangial cells (GMCs) are crucial in sustaining the structural integrity and filtration efficiency of the renal glomerulus [1]. However, their senescence has emerged as an important mediator of multiple age‐associated kidney pathologies, such as diabetic nephropathy, chronic kidney disease, and glomerulosclerosis [2, 3]. Senescent GMCs secrete a repertoire of pro‐inflammatory factors and matrix‐remodeling enzymes, collectively termed the senescence‐associated secretory phenotype (SASP), which promotes extracellular matrix (ECM) deposition, glomerular fibrosis, and impaired renal function [4]. In addition, these senescent cells exhibit reduced reparative capacity and contribute to disruption of the glomerular filtration barrier [5]. Therefore, elucidating the molecular mechanisms underlying GMC senescence may offer novel insights into the renal aging processes and might offer promising treatment targets for preventing or mitigating glomerular fibrosis and associated pathologies.

Long non‐coding RNAs (lncRNAs) have recently been identified as crucial regulators in the pathogenesis of kidney diseases and aging processes [6]. LncRNAs participate in epigenetic modulation, inflammatory responses, cell cycle regulation, and fibrosis [7]. Accumulating evidence suggests that dysregulated expression of certain lncRNAs has been implicated in promoting renal cell senescence [8]. For instance, opa interacting protein 5 antisense RNA 1 (OIP5‐AS1) expression is elevated in hydrogen peroxide‐induced senescent human umbilical vein endothelial cells, while its silencing alleviates endothelial senescence, suggesting a pro‐senescent role [9]. Moreover, OIP5‐AS1 has also been reported to facilitate epithelial‐to‐mesenchymal transition (EMT) and enhance renal fibrotic responses under high‐glucose conditions in human kidney cells [10]. However, the precise role of OIP5‐AS1 in the senescence of GMCs has not been fully elucidated. Our previous studies demonstrated that guiqi dingnian prescription (GDP), a traditional Chinese medicine formula, alleviates renal aging by inhibiting STAT1/STAT3 signaling in a mouse model [11]. Notably, an evident elevation of OIP5‐AS1 expression was observed in the kidneys of d‐galactose (d‐gal)‐treated aging mice, and GDP treatment effectively suppressed its expression (Supporting Information Figure S1). These observations imply that OIP5‐AS1 may participates in regulation of GMC senescence, and comprehensive exploration of its molecular basis and biological significance may yield novel perspectives for developing targeted interventions in renal aging.

Mechanistically, lncRNAs typically function by interacting with RNA‐binding proteins, thereby influencing mRNA stability, localization, and translation [12]. Embryonic lethal abnormal vision‐like RNA‐binding protein 1 (ELAVL1, also known as HuR) is a widely expressed RBP that has been implicated in diverse cellular events such as cell growth, inflammatory response, and aging [13]. Earlier studies indicated that reducing ELAVL1 expression suppresses cardiac senescence [14], and lncRNA RMRP exacerbates renal injury in acute kidney injury models by enhancing COX2 expression via binding to ELAVL1 [15]. This suggests that ELAVL1 potentially participates in the modulation of GMC senescence. Furthermore, ENCORI (https://rnasysu.com/encori/) prediction indicates a potential binding relationship between lncRNA OIP5‐AS1 and ELAVL1.

High‐mobility group box 1 (HMGB1), a nuclear protein that also acts as a damage‐associated molecular pattern (DAMP) factor, is recognized as an essential mediator of both cellular senescence and inflammatory responses. It plays essential roles in neural aging [16], intervertebral disc degeneration [17], and cardiac senescence [18]. Notably, accumulation of HMGB1 in GMCs activates the NF‐κB signaling cascade, thereby accelerating the pathological progression of diabetic nephropathy [19]. Moreover, ENCORI analysis further supports the possibility of a direct interaction between ELAVL1 and HMGB1 mRNA.

Based on these observations, we hypothesize that lncRNA OIP5‐AS1 promotes GMC senescence by binding to ELAVL1 and enhancing the stability and expression of HMGB1. This study aims to elucidate the OIP5‐AS1/ELAVL1/HMGB1 axis in GMC senescence and provide new insights into potential therapeutic strategies targeting renal aging and fibrosis.

## 2 Methods

### 2.1 Animal Model and Treatment

Eight‐week‐old male C57BL/6 J mice (20 ± 2 g) were procured from Beijing Vital River Laboratory Animal Technology Co. Ltd. (Beijing, China), and maintained under standard housing conditions. To induce aging, the animals were randomly allocated into three groups (*n* = 6 each): control, d‐gal, and d‐gal combined with high‐dose GDP. d‐gal was administered subcutaneously at 120 mg/kg/day for 42 consecutive days. Starting from day 21, GDP solution was administered by gavage at 8.32 g/kg/day for GDP group, equivalent to twice the human dose adjusted for body surface area. All animal procedures received approval from the ethics committee of Yili Normal University (YLSFDWLL202403317).

### 2.2 Cell Culture and Senescence Induction

Mouse GMCs (SV40‐MES13; RRID: CVCL_5368) were obtained from Procell Life Science & Technology Co. Ltd. (Wuhan, China; Cat. No. CL‐0470). The cells were confirmed to be free of mycoplasma contamination by PCR‐based testing before use. Cells were maintained in DMEM (Gibco, Thermo Fisher Scientific, USA) supplemented with 10% fetal bovine serum (FBS; Gibco, USA) at 37°C in a 5% CO_2_ humidified incubator. To induce cellular senescence, cells at 60%–70% confluence were exposed to d‐gal (5–45 g/L; Sigma‐Aldrich, USA) for 48 h, and cellular metabolic activity was evaluated using the CCK‐8 assay (Beyotime, China) according to the manufacturer's protocol.

### 2.3 RNA Interference and Plasmid Transfection

Three short hairpin RNA (sh‐RNAs) targeting OIP5‐AS1 and ELAVL1, together with their respective negative controls (sh‐NC), were synthesized by GeneChem (Shanghai, China). SV40‐MES13 cells were seeded into 6‐well plates and cultured to approximately 60%–70% confluence prior to transfection. Cells were transfected using Lipofectamine 3000 (Thermo Fisher Scientific, USA; Cat# L3000015) according to the manufacturer's instructions. For each well, 2 μg shRNA plasmid or 1.5 μg overexpression plasmid was used. Overexpression plasmids for ELAVL1 and HMGB1 (oe‑ELAVL1 and oe‑HMGB1) and their empty vector controls (oe‑NC) were transfected in the same manner. The culture medium was replaced with fresh complete medium after 24 h, and cells were harvested 48 h after transfection for subsequent experiments. Three shRNAs targeting OIP5‐AS1 and ELAVL1, together with sh‐NC, were constructed using the PLKO.1‐PURO vector (derived from HIV‐1) with the U6 promoter, while oe‑ELAVL1, oe‑HMGB1, and oe‑NC were constructed using the pLV3‐CMV‐3×FLAG‐MCS‐EF1a‐mCherry‐Puro vector, an HIV‐1‐based lentiviral transfer vector driven by the CMV promoter. Detailed sequence information is provided in Supporting Information Table S1.

### 2.4 Reverse Transcription Quantitative Polymerase Chain Reaction (RT‐qPCR)

Total RNA was isolated from cultured cells and tissue samples using TRIzol reagent (Invitrogen, USA). cDNA was synthesized from 1 μg total RNA using a reverse transcription kit (Takara, Japan) according to the manufacturer's instructions with random hexamer and oligo(dT) primers. qPCR was performed with SYBR Green Master Mix (Thermo Fisher Scientific, USA) on a QuantStudio 6 Flex Real‐Time PCR System (Applied Biosystems, USA). GAPDH served as the internal control, and relative gene expression was analyzed by the 2^−ΔΔCt^ method. The primer sequences used in this study are shown in Table 1.

**Table 1:** The primers used in the present study.

Name	Forward primer (5′–3′)	Reverse primer (5′–3′)
OIP5‐AS1	AAGCACAGTTGACCGCAGTA	CCAACCCAGTCTCACATGCT
ELAVL1	ACTGAACGGCTTGAGACTCCAG	CCACATCCTTCTGTGTCATGGTC
HMGB1	CCAAGAAGTGCTCAGAGAGGTG	GTCCTTGAACTTCTTTTTGGTCTC
GAPDH	CATCACTGCCACCCAGAAGACTG	ATGCCAGTGAGCTTCCCGTTCAG
JPX	ACTTATAAAATGGCGGCGTC	TCACCAGGGCATGTTCATTA
MIR22HG	AAGCAGATGTGGCTGAAACC	TCACAATGGTTGGTACTGCC
U6	CGCACTTTACGGCTACCTCT	GCGACAAGGGAAGGGAACAA

### 2.5 Western Blot (WB)

Total proteins were extracted using RIPA lysis buffer (Beyotime, China) supplemented with protease inhibitors (Roche, Switzerland). Protein concentrations were determined using a BCA Protein Assay Kit (Beyotime, China) according to the manufacturer's instructions. Equal amounts of protein (25 μg per lane) were resolved by SDS–PAGE and transferred to PVDF membranes (Millipore, USA). After blocking with 5% skim milk in TBST for 1 h at room temperature, the membranes were incubated overnight at 4°C with primary antibodies against p16 (Thermo Fisher Scientific, USA, MA5‐17142; 1:1000), p21 (Thermo Fisher Scientific, USA, MA5‐14949; 1:1000), and p53 (Thermo Fisher Scientific, USA, AH00152; 1:500), HMGB1 (Invitrogen, USA, MA5‑17278; 1:1000), ELAVL1/HuR (ProteinTech, China, 11910‑1‑AP; 1:3000), and β‐actin (Invitrogen, USA, MA5‐15452; 1:1000). Following washing, the membranes were incubated with HRP‐conjugated secondary antibodies (ab6721 or ab6728, Abcam, UK; 1:10,000) at room temperature for 1 h. Protein signals were detected by enhanced chemiluminescence (ECL; Thermo Fisher Scientific, USA). Band intensities were quantified using ImageJ software (NIH, USA; version 1.53), and target protein expression levels were normalized to β‐actin.

### 2.6 Senescence‐Associated β‐Galactosidase (SA‐β‐gal) Staining

Cellular senescence was assessed by a SA‑β‑gal staining kit (Beyotime, China; C0603). After fixation, samples were incubated with X‑gal solution overnight at 37°C. Blue‐stained senescent cells were observed under a bright‐field microscope and quantified in five random fields per sample.

### 2.7 Enzyme‐Linked Immunosorbent Assay (ELISA)

The levels of SASP‐associated cytokines in cell culture supernatants were determined with commercial ELISA kits (IL‐6: E‐EL‐M0044; TNF‐α: E‐EL‐M3063; IL‐1β: E‐EL‐M0037; IL‐18: E‐EL‐M0730; Elabscience, China) in accordance with the manufacturer's instructions. Cell culture supernatants were collected after 48 h of d‐gal treatment under complete culture medium containing 10% fetal bovine serum and centrifuged at 3000 rpm for 10 min to remove cellular debris before analysis. Absorbance was recorded at 450 nm with a microplate reader (Bio‐Rad, USA). The 48 h time point was selected based on preliminary experiments and previous studies showing that d‐gal exposure within this duration is sufficient to induce senescence‐associated phenotypes and inflammatory responses [20].

### 2.8 RNA Pull‐Down Assay

Biotin‐labeled sense and antisense RNA probes for OIP5‑AS1 or HMGB1 were generated by in vitro transcription using PCR‐amplified DNA templates containing the T7 promoter sequence with the T7 transcription system (Roche, Switzerland) in the presence of Biotin‐UTP. The antisense probes were used as negative controls to exclude nonspecific RNA–protein interactions. SV40‐MES13 cells were treated with d‐gal for 48 h before collection. Cell lysates were then incubated with RNA probes and streptavidin‐conjugated magnetic beads (Invitrogen, USA). After washing, bound proteins were analyzed by WB to detect ELAVL1. The template sequences and primer information are provided in Supporting Information Table S2.

### 2.9 RNA Immunoprecipitation (RIP)

RIP assay was conducted with the Magna RIP Kit (Millipore, USA) following the manufacturer's instructions. SV40‐MES13 cells were treated with d‐gal for 48 h before lysis for RIP experiments. Cell lysates were incubated with an anti‐ELAVL1 antibody (Proteintech, China; 11910‐1‐AP; 5 μg) or normal IgG (Millipore, USA; 12‐370; 5 μg) as a negative control. The immunoprecipitated RNA was purified and analyzed by RT‐qPCR to determine the enrichment of OIP5‐AS1 and HMGB1.

### 2.10 Actinomycin D Transcriptional Stability Assay

To assess HMGB1 mRNA stability, SV40‑MES13 cells were first treated with d‐gal for 48 h to induce senescence. Cells were then exposed to actinomycin D (5 µg/mL; Sigma‐Aldrich, USA) for transcription inhibition. Cells were harvested at 0, 3, and 6 h post‐treatment. Extracted RNA was analyzed by RT‐qPCR to determine HMGB1 transcript decay rates.

### 2.11 Statistical Analysis

Data are expressed as mean ± SD. Analyzes were performed using GraphPad Prism 10.1.2 (USA). An unpaired Student's t‐test was used for two‐group analyzes, and one‐way ANOVA with Tukey's post hoc correction was employed for multiple comparisons. Statistical significance was defined as *p* < 0.05.

## 3 Results

### 3.1 OIP5‐AS1 Expression Is Upregulated in d‐gal‐Induced Senescent GMCs

**Figure 1:**
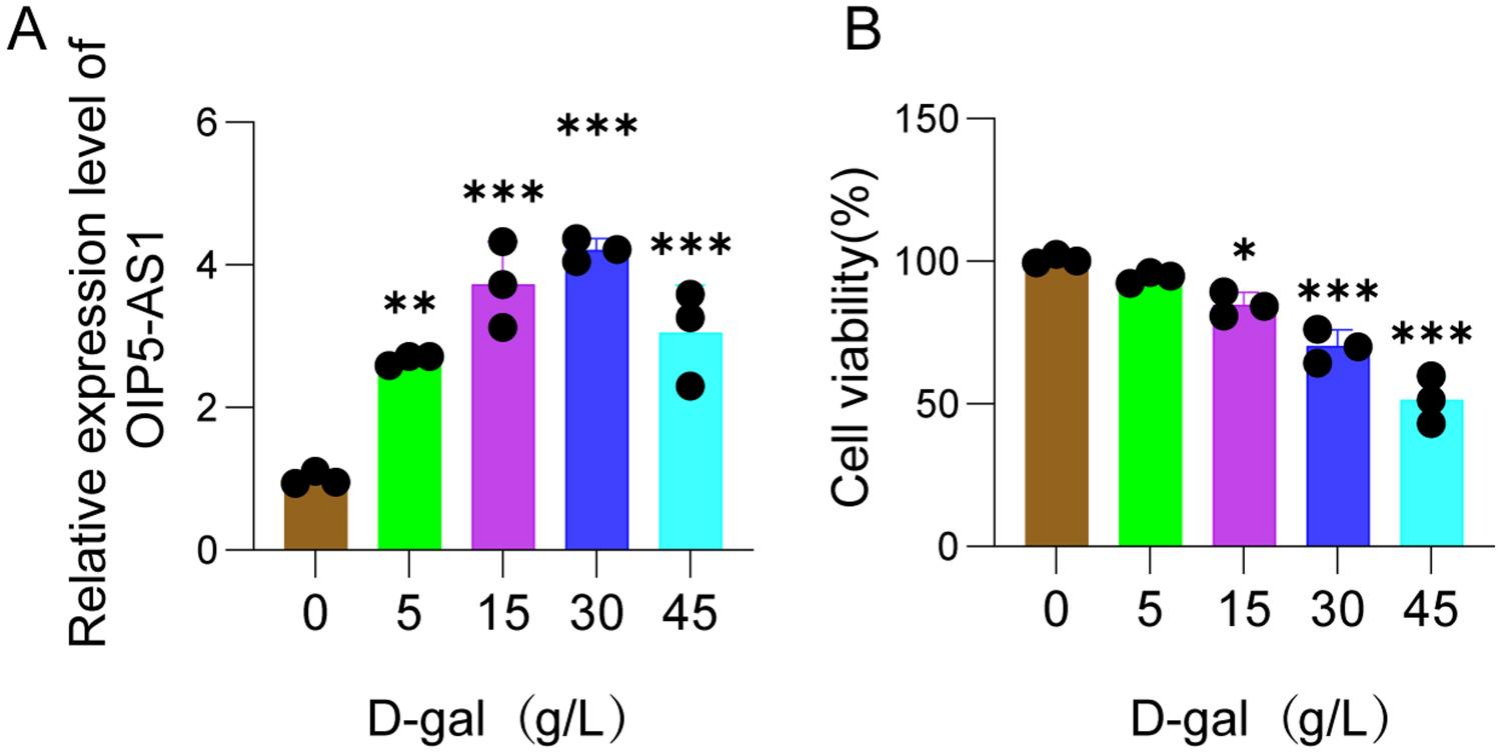
OIP5‐AS1 expression is upregulated in d‐gal‐induced senescent GMCs. (A) RT‐qPCR analysis of OIP5‐AS1 level in SV40‐MES13 cells treated with various concentrations of d‐gal for 48 h. (B) Cellular metabolic activity assessment using CCK‐8 assay after d‐gal treatment. *n* = 3, *p < 0.05, **p < 0.01, ***p < 0.001.

To explore the involvement of OIP5‐AS1 in cellular senescence, mouse GMCs (SV40‐MES13) were treated with increasing concentrations of d‐gal. RT‐qPCR analysis demonstrated a dose‐dependent elevation of OIP5‐AS1 level when d‐gal concentrations ranged from 5 to 30 g/L (Figure 1A). At 45 g/L, OIP5‐AS1 expression began to decline, coinciding with a marked reduction in cellular metabolic activity (< 50%) as assessed using the CCK‐8 assay (Figure 1B). Based on these findings, 30 g/L d‐gal was selected for subsequent experiments to induce senescence without excessive cytotoxicity.

### 3.2 Knockdown of OIP5‐AS1 Attenuates d‐gal‐Induced GMCs Senescence

**Figure 2:**
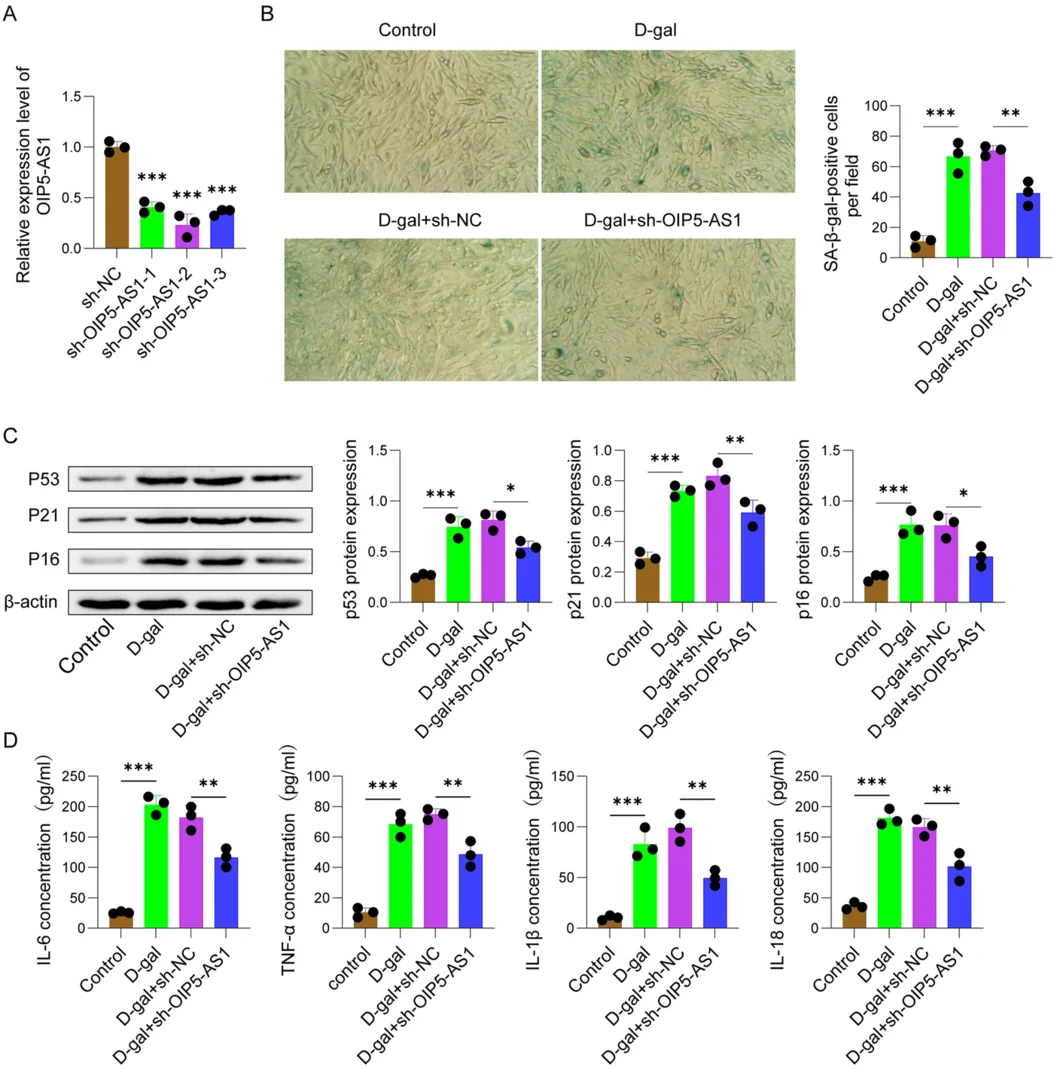
Knockdown of OIP5‐AS1 suppresses d‐gal‐induced cellular senescence. (A) RT‐qPCR screening of three sh‐RNAs targeting OIP5‐AS1 to determine knockdown efficiency. (B) SA‐β‐gal staining to evaluate senescent cell proportion in different groups. (C) WB analysis of senescence markers p16, p21, and p53. (D) ELISA measurement of SASP cytokines IL‐6, TNF‐α, IL‐1β, and IL‐18 in cell culture supernatants. *n* = 3, *p < 0.05, **p < 0.01, ***p < 0.001.

To clarify the functional significance of OIP5‐AS1 in mesangial cell senescence, three specific sh‐RNAs targeting OIP5‐AS1 were screened. Among them, sh‐OIP5‐AS1‐2 exhibited the most efficient knockdown and was used for subsequent experiments (Figure 2A). d‐gal exposure markedly enhanced SA‐β‐gal staining and elevated the protein levels of p16, p21, and p53, whereas OIP5‐AS1 knockdown mitigated these effects (Figure 2B–C). ELISA results further demonstrated that OIP5‐AS1 silencing significantly suppressed SASP cytokine secretion (IL‐6, IL‐1β, TNF‐α, IL‐18) (Figure 2D). These results suggest that OIP5‐AS1 promotes mesangial cell senescence and inflammatory responses.

### 3.3 OIP5‐AS1 Upregulates HMGB1 Expression Through Interaction With ELAVL1

**Figure 3:**
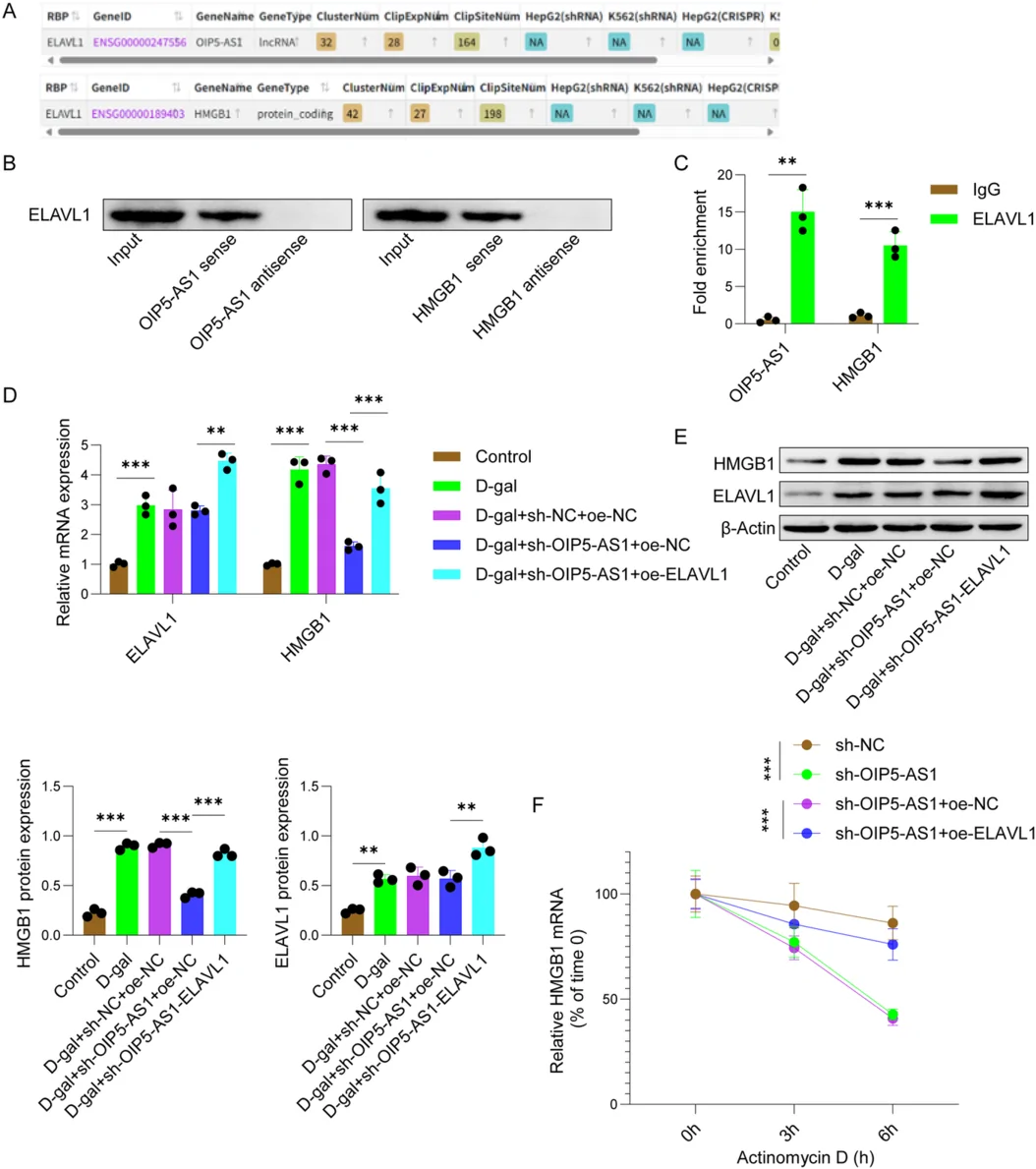
OIP5‐AS1 upregulates HMGB1 expression by interacting with ELAVL1. (A) ENCORI database prediction showing potential binding interactions among OIP5‐AS1, ELAVL1, and HMGB1. (B) RNA pull‐down assay verifying the binding of ELAVL1 and OIP5‐AS1/HMGB1. (C) RIP assay confirming ELAVL1 binding to OIP5‐AS1 and HMGB1 in SV40‐MES13 cells. (D) RT‐qPCR detection of ELAVL1 and HMGB1 mRNA levels following OIP5‐AS1 knockdown and ELAVL1 overexpression. (E) WB detection of ELAVL1 and HMGB1 protein levels under the same conditions. (F) Actinomycin D transcriptional inhibition assay for HMGB1 mRNA stability. *n* = 3, **p < 0.01, ***p < 0.001.

To elucidate the downstream mechanism of OIP5‐AS1 in promoting senescence, ENCORI database (https://rnasysu.com/encori/) analysis predicted potential binding sites between OIP5‐AS1 and the RNA‐binding protein ELAVL1, as well as between ELAVL1 and HMGB1 mRNA (Figure 3A). RNA pull‐down and RIP assays confirmed the interactions of ELAVL1 with both OIP5‐AS1 and HMGB1(Figure 3B–C). In the d‐gal group, ELAVL1 and HMGB1 expression levels were significantly increased. Knockdown of OIP5‐AS1 suppressed HMGB1 without affecting ELAVL1. On this basis, overexpression of ELAVL1 rescued HMGB1 expression (Figure 3D–E). Furthermore, an actinomycin D transcriptional inhibition assay showed that OIP5‐AS1 knockdown significantly reduced HMGB1 mRNA stability, which was restored by ELAVL1 overexpression (Figure 3F). These findings indicate that OIP5‐AS1 enhances HMGB1 expression by stabilizing its mRNA via ELAVL1 interaction.

### 3.4 The OIP5‐AS1/ELAVL1 Axis Promotes GMC Senescence

**Figure 4:**
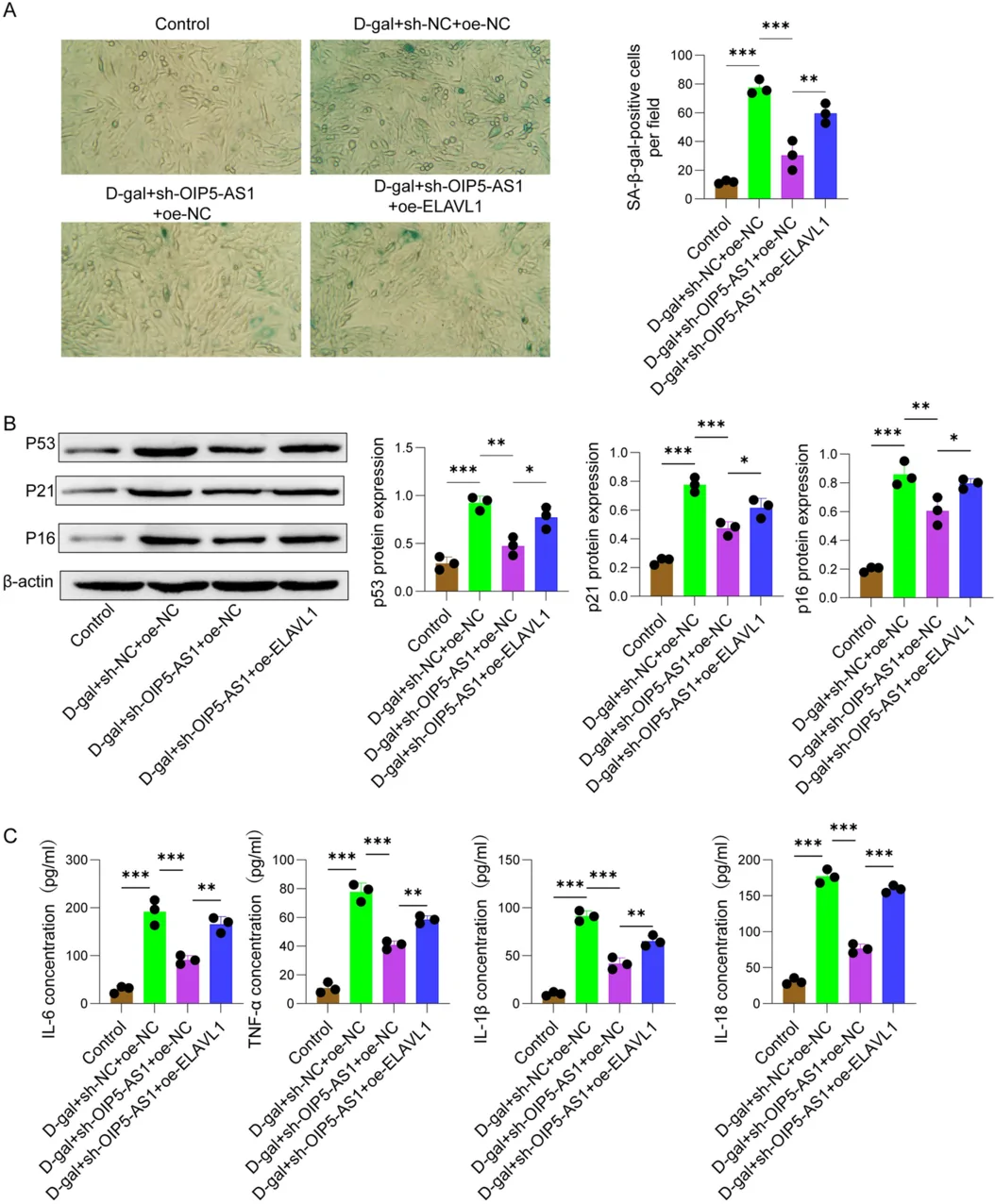
The OIP5‐AS1/ELAVL1 interaction promotes GMC senescence. (A) SA‐β‐gal staining to assess senescent cell proportion under OIP5‐AS1 knockdown and ELAVL1 overexpression. (B) WB analysis of senescence‐associated proteins p16, p21, and p53. (C) ELISA quantification of SASP cytokines IL‐6, TNF‐α, IL‐1β, and IL‐18. *n* = 3, *p < 0.05, **p < 0.01, ***p < 0.001.

To further validate the functional role of the OIP5‐AS1/ELAVL1 axis, we examined cellular senescence in d‐gal‐treated cells following OIP5‐AS1 knockdown and ELAVL1 overexpression. Silencing OIP5‐AS1 led to a pronounced reduction in SA‐β‐gal‐positive cells and downregulation of p16, p21, and p53, effects that were reversed upon ELAVL1 overexpression (Figure 4A–B). In addition, ELISA revealed that overexpression of ELAVL1 restored the secretion of SASP‐associated cytokines, including IL‐6, TNF‐α, IL‐1β, and IL‐18 (Figure 4C). These data support the notion that OIP5‐AS1 promotes mesangial cell senescence via interaction with ELAVL1.

### 3.5 The ELAVL1/HMGB1 Axis Mediates OIP5‐AS1‐Induced Senescence

**Figure 5:**
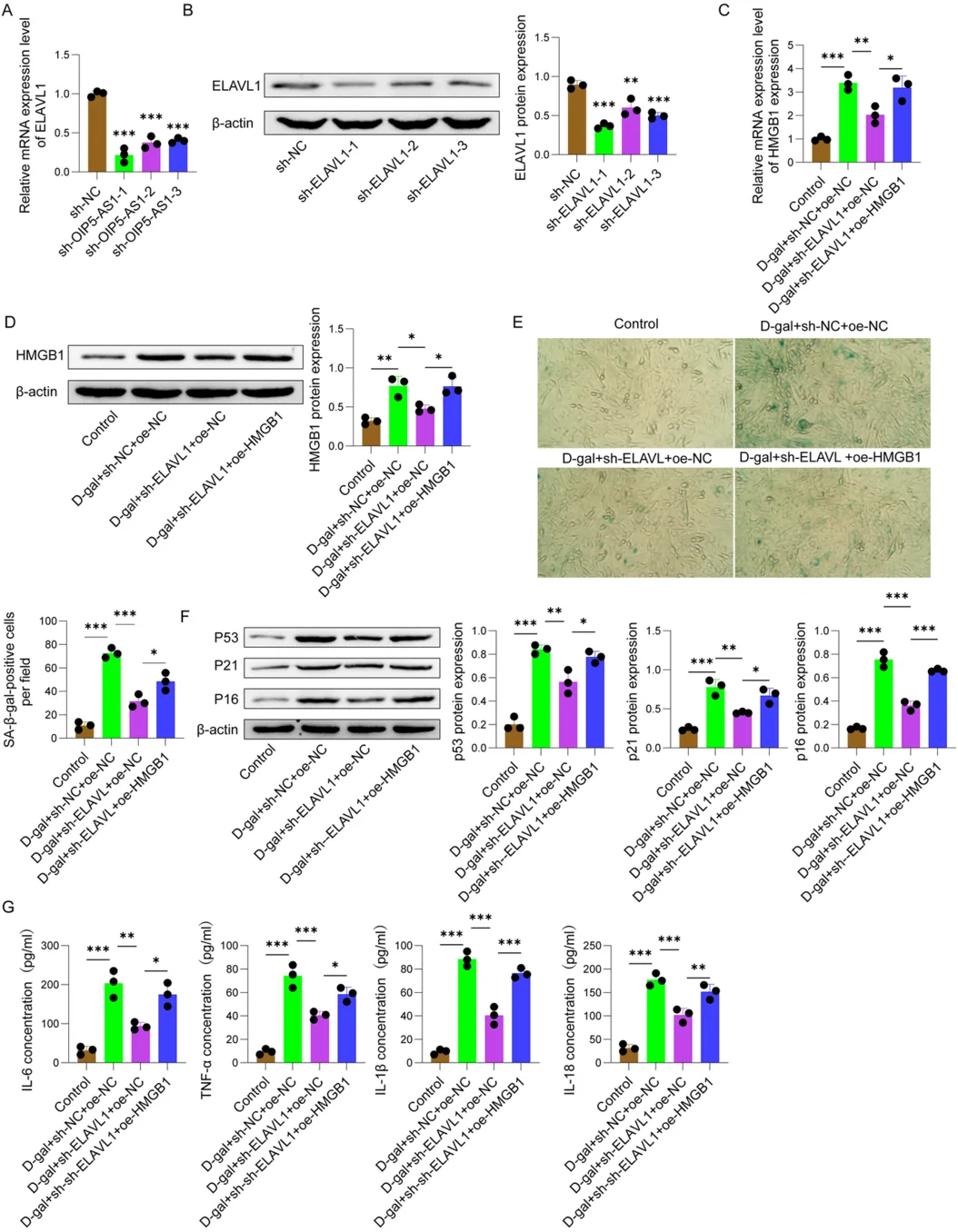
The ELAVL1/HMGB1 axis mediates OIP5‐AS1‐induced mesangial cell senescence. (A) RT‐qPCR screening of three sh‐RNAs targeting ELAVL1 to determine knockdown efficiency. (B) WB validation of ELAVL1 knockdown efficiency. (C) RT‐qPCR detection of HMGB1 mRNA expression after ELAVL1 knockdown and HMGB1 overexpression. (D) WB detection of HMGB1 protein levels under different conditions. (E) SA‐β‐gal staining to evaluate senescent cell proportion. (F) WB analysis of senescence markers p16, p21, and p53. (G) ELISA detection of SASP cytokines IL‐6, TNF‐α, IL‐1β, and IL‐18 across treatment groups. *n* = 3, *p < 0.05, **p < 0.01, ***p < 0.001.

To further investigate the role of the ELAVL1/HMGB1 axis in mesangial cell senescence, three sh‐RNAs were designed to target ELAVL1, among which sh‐ELAVL1‐1 achieved the greatest knockdown efficiency and was selected for subsequent experiments (Figure 5A–B). ELAVL1 knockdown significantly decreased HMGB1 mRNA and protein levels, as confirmed by RT‐qPCR and WB, whereas HMGB1 overexpression restored its expression (Figure 5C–D). Functionally, ELAVL1 knockdown alleviated cellular senescence, evidenced by fewer SA‐β‐gal‐positive cells, reduced levels of p16, p21, and p53, and decreased secretion of inflammatory cytokines (IL‐6, TNF‐α, IL‐1β, and IL‐18) (Figure 5E–G). These inhibitory effects were reversed by HMGB1 overexpression, supporting that ELAVL1 promotes mesangial cell senescence through HMGB1.

## 4 Discussion

Age‐related kidney diseases, such as glomerulosclerosis and diabetic nephropathy, are tightly linked to GMC senescence [21]. However, the upstream regulatory mechanisms driving GMC aging remain poorly understood. In this work, we identified a novel OIP5‐AS1/ELAVL1/HMGB1 axis that promotes GMC senescence, providing new mechanistic insights into renal aging.

Emerging evidence has emphasized the pivotal role of OIP5‐AS1 in cellular senescence. Its expression is significantly upregulated in hydrogen peroxide‐induced senescent endothelial cells, and silencing OIP5‐AS1 delays premature aging by reducing ROS accumulation and oxidative damage [9]. It also activates the p53 pathway to promote neuronal senescence and enhances STAT3 or NF‐κB–mediated inflammation, thereby facilitating stress‐induced aging and SASP release [22]. Consistent with these findings, our previous study showed marked OIP5‐AS1 upregulation in the kidneys of d‐gal–induced senescent mice, which was effectively suppressed by GDP (Supporting Information Figure S1). In the present study, we confirmed these observations in GMCs: OIP5‐AS1 was markedly upregulated in d‐gal–treated cells, whereas OIP5‐AS1 silencing attenuated senescence‐related features, characterized by decreased SA‐β‐gal staining and reduced expression of p16, p21, and p53, and decreased SASP secretion. Collectively, these results identify OIP5‐AS1 as a pro‐senescence factor in renal cells.

The diverse functions of lncRNAs are frequently mediated through their interactions with RBPs, which influence mRNA fate and cellular responses [23]. ELAVL1 (HuR) regulates the stability and translation of multiple transcripts involved in cellular senescence and inflammation, including p21 and p16, as well as pro‐inflammatory mediators such as TNF‐α and IL‐6 [13]. In our study, RNA pull‐down and RIP assays confirmed a direct interaction between OIP5‐AS1 and ELAVL1, which stabilizes HMGB1 mRNA. Thus, OIP5‐AS1 likely functions as a molecular scaffold, recruiting ELAVL1 to enhance HMGB1 expression and activate senescence‐associated pathways.

HMGB1 is normally a nuclear protein that regulates DNA architecture and gene expression under homeostatic conditions but translocates under stress to act as a DAMP, triggering inflammatory cascades and accelerating renal fibrosis and diabetic nephropathy [24, 25]. Elevated HMGB1 levels have been reported in various age‐related disorders, including neurodegeneration [26], cardiovascular injury, and intervertebral disc degeneration [27]. Previous studies have reported that cytoplasmic HMGB1 accumulation in the kidney is associated with diabetic nephropathy progression and glomerular fibrosis [19]. However, the regulatory role of this pathway in GMC senescence remains largely unexplored. In this study, we identified a potential OIP5‑AS1/ELAVL1/HMGB1 regulatory axis in d‐gal–induced GMCs. Specifically, OIP5‑AS1 knockdown was found to reduce HMGB1 mRNA stability and protein expression, thereby suppressing SASP secretion and senescence markers. Moreover, ELAVL1 overexpression partially restored HMGB1 levels and reversed these effects, whereas HMGB1 overexpression rescued the phenotype caused by ELAVL1 silencing.

In summary, these results shed new light on the molecular basis of renal aging and indicate that targeting the OIP5‐AS1/ELAVL1/HMGB1 axis may offer a promising strategy to delay glomerular aging and related pathologies. However, several limitations should be noted. First, all mechanistic investigations were performed in vitro using a d‐gal‐induced cell model, which may not fully replicate in vivo conditions. Second, although ELAVL1‐mediated mRNA stabilization was confirmed, potential contributions from transcriptional regulation or epigenetic modifications of HMGB1 were not excluded. Third, OIP5‐AS1 may also promote GMC senescence via interactions with other RNA‐binding proteins or microRNAs, which warrants further exploration. Fourth, to maintain consistency with our in vivo experimental model (Supporting Information Figure S1), we selected mouse glomerular mesangial cells (SV40‐MES13) for this study. Although the present study was performed primarily in SV40‐MES13—which are immortalized by SV40 large T antigen and therefore may not fully reflect the characteristics of primary cells—these cells can still develop senescence‐associated phenotypes under stress stimulation and are widely used in renal senescence research [28, 29]. Future studies using human GMCs (such as HRMCs) and clinical renal samples are needed to further validate the clinical relevance of the OIP5‐AS1/ELAVL1/HMGB1 axis.

## 5 Conclusions

**Figure 6:**
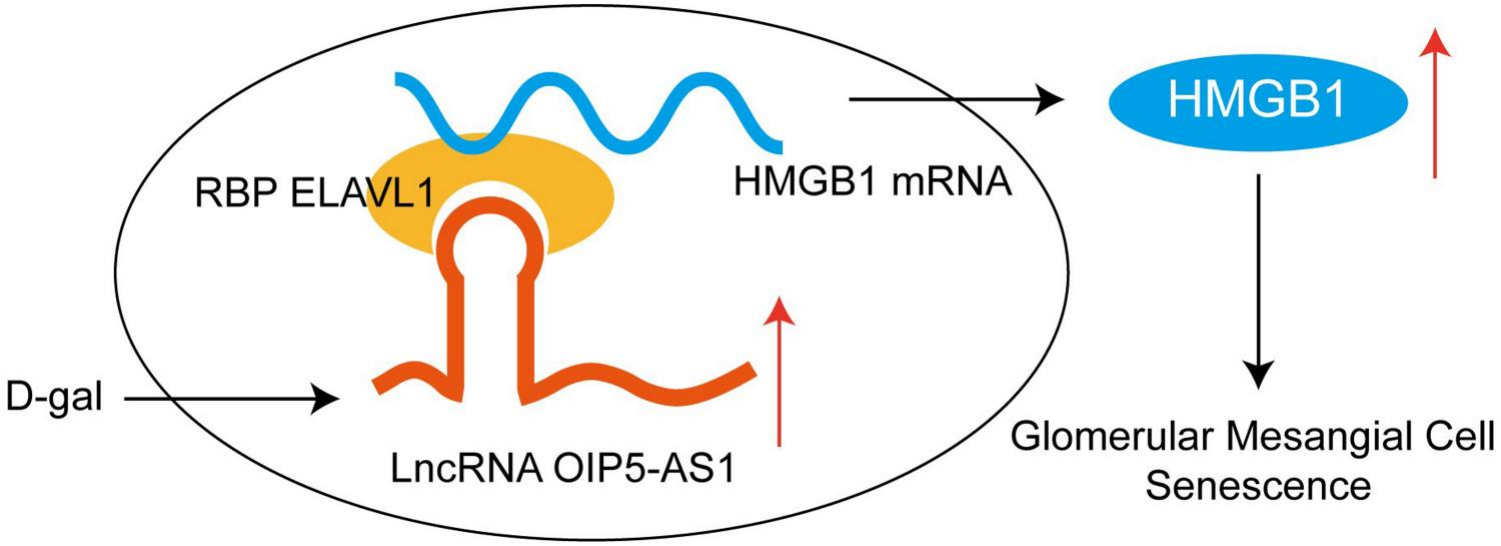
Schematic illustration of the OIP5‐AS1/ELAVL1/HMGB1 axis in d‐gal‐induced GMC senescence. d‐gal treatment upregulates OIP5‐AS1 expression in GMCs. OIP5‐AS1 interacts with ELAVL1 to enhance the stability of HMGB1 mRNA, thereby increasing HMGB1 expression and promoting senescence‐associated phenotypes.

This work reveals that the lncRNA OIP5‐AS1 promotes GMC senescence via the ELAVL1/HMGB1 axis. OIP5‐AS1 interacts with ELAVL1 to stabilize HMGB1 mRNA, leading to increased HMGB1 expression and senescence marker activation. Knockdown of OIP5‐AS1 alleviates senescence and reduces SASP secretion. These findings suggest that targeting the OIP5‐AS1/ELAVL1/HMGB1 pathway may offer a potential therapeutic approach to delay renal aging and related fibrosis. A schematic illustration of the proposed mechanism is presented in Figure 6.

## Author Contributions

Zhenxin Ren, Huijun Cheng and Zhiwei Wu designed the study and literature research. Hengcai Hu, Li Sun and Zhiwei Wu defined the intellectual content. Zhenxin Ren, Hengcai Hu and Li Sun performed experiment. Li Sun and Na Xue collected the data. Huijun Cheng, Li Sun and Na Xue analyzed the data. All authors reviewed the article.

## Ethics Statement

All animal procedures received approval from the ethics committee of Yili Normal University (YLSFDWLL202403317).

## Conflicts of Interest

The authors declare that they have no known competing financial interests or personal relationships that could have appeared to influence the work reported in this article.

## Supporting information


Supporting File 1

Supporting File 2

## Data Availability

The data that support the findings of this study are available from the corresponding author upon reasonable request. The datasets used or analyzed during the current study are available from the corresponding author on reasonable request.
