# Supplementary material for: LncRNA OIP5‐AS1 Promotes Glomerular Mesangial Cell Senescence by Interacting With ELAVL1 to Upregulate HMGB1
Source: Cell Biochem Funct. 2026 Sep 20;44(9):e70311. doi: 10.1002/cbf.70311 (PMC13591199; doi:10.1002/cbf.70311)
Supplement: Supplementary file 1 [file CBF-44-e70311-s001.docx]

**Supplementary Table 1. Primer sequence information**

|  | Sequence information |
| --- | --- |
| sh-OIP5-AS1-1 | Forward: 5'-CCGG-GCAGTAAGTTCCAAATATTTA-CTCGAG-TAAATATTTGGAACTTACTGC-TTTTTG-3'  Reverse: 5'-AATTCAAAAA-GCAGTAAGTTCCAAATATTTA-CTCGAG-TAAATATTTGGAACTTACTGC-3' |
| sh-OIP5-AS1-2 | Forward: 5'-CCGG-GAAGGCTTTGGACAGTTTAAT-CTCGAG-ATTAAACTGTCCAAAGCCTTC-TTTTTG-3'  Reverse: 5'-AATTCAAAAA-GAAGGCTTTGGACAGTTTAAT-CTCGAG-ATTAAACTGTCCAAAGCCTTC-3' |
| sh-OIP5-AS1-3 | Forward: 5'-CCGG-GGACCGTGTGAGGAACCATAT-CTCGAG-ATATGGTTCCTCACACGGTCC-TTTTTG-3'  Reverse: 5'-AATTCAAAAA-GGACCGTGTGAGGAACCATAT-CTCGAG-ATATGGTTCCTCACACGGTCC-3' |
| sh-ELAVL1-1 | Forward: 5'-CCGG-GCCAATCCCAACCAGAACAAA-CTCGAG-TTTGTTCTGGTTGGGATTGGC-TTTTTG-3'  Reverse: 5'-AATTCAAAAA-GCCAATCCCAACCAGAACAAA-CTCGAG-TTTGTTCTGGTTGGGATTGGC-3' |
| sh-ELAVL1-2 | Forward: 5'-CCGG-CGAGGTTGAATCTGCAAAGCT-CTCGAG-AGCTTTGCAGATTCAACCTCG-TTTTTG-3'  Reverse: 5'-AATTCAAAAA-CGAGGTTGAATCTGCAAAGCT-CTCGAG-AGCTTTGCAGATTCAACCTCG-3' |
| sh-ELAVL1-3 | Forward: 5'-CCGG-CCCACAAATGTTAGACCAATT-CTCGAG-AATTGGTCTAACATTTGTGGG-TTTTTG-3'  Reverse: 5'-AATTCAAAAA-CCCACAAATGTTAGACCAATT-CTCGAG-AATTGGTCTAACATTTGTGGG-3' |
| sh-NC | Forward: 5'-CCGG-GCGCGATAGCGCTAATAATTT-CTCGAG-AAATTATTAGCGCTATCGCGC-TTTTTG-3'  Reverse: 5'-AATTCAAAAA-GCGCGATAGCGCTAATAATTT-CTCGAG-AAATTATTAGCGCTATCGCGC-3' |
| oe-ELAVL1 | Forward: 5'-ACCTCCATAGAAGATTCTAGAATGTCTAATGGTTATGAAGACCACATGGC-3'  Reverse: 5'-TTGTAGTCCATGGTGGCGAATTCTTATTTGTGGGACTTGTTG-3' |

**Supplementary Table 2. Information generated by RNA pull-down probe**

|  | Sequence information |
| --- | --- |
| Template sequences | GTTTTTTTTTTAATGTCTTCCAATTTTAACACTATTATAAGCACAGTTGACCGCAGTAAGTTCCAAATATTTAATATAATTTGAAGTGAAAGAAATGTTATCTAAATTCTGGCTCACTCTTTGACAGTTTTCCTGGACAAAAACAAGATACGATAAAAGCAGAAACCAGGTTTGATTTTGTATGGGAGTGAAATCATTACAGTTTTATGGGTATAAATCTACATTTTTAAACAAGATTTTTCTCTT |
| OIP5-AS1 sense | Forward-T7: 5'-TAATACGACTCACTATAGGGCACTATTATAAGCACAGTTGACCG-3'  Reverse：5'-AAGAGAAAAATCTTGTTTAAAAATGT-3' |
| OIP5-AS1 antisense | Forward： 5'-CACTATTATAAGCACAGTTGACCG-3'  Reverse -T7: 5'-TAATACGACTCACTATAGGGAAGAGAAAAATCTTGTTTAAAAATGT-3' |
